# The Unexplored Benefits of Paediatric Cardiac Humanitarian Work in the Developing World

**DOI:** 10.3389/fped.2021.555085

**Published:** 2021-07-06

**Authors:** Sahil Nichani, Sanjiv Nichani

**Affiliations:** ^1^Barts and the London School of Medicine, Queen Mary University, Whitechapel, United Kingdom; ^2^Leicester Children's Hospital and East Midlands Congenital Heart Centre, Leicester, United Kingdom

**Keywords:** congenital heart defect, paediatric cardiac surgery, humanitarian, developing countries, charity, intensive care, cardiac surgeon

## Introduction

Congenital heart disease is the most common type of birth defect with an average incidence of ~9.0 per 1,000 live births (1). There are 1.4 million children born with congenital heart disease in the world every year with over a million of these children being born in low and middle income countries (LMIC). As the rate of infectious diseases has fallen by 50% since 1990, birth defects have become the 4th highest cause of childhood mortality in the world (1). In response to this, there has been a proliferation of teams from across the developed world that visit various LMIC to perform cardiac surgery on children and in certain instances to help build capacity for paediatric cardiac services. Whilst these lifesaving endeavours are laudable and well-documented, what has not been explored are the significant mutual benefits accrued to the visiting international teams and local teams during these missions. Typically, each international team is composed of the same personnel required to carry out paediatric cardiac surgery in their home countries which therefore means a paediatric cardiologist, paediatric cardiac anaesthetist, paediatric cardiac surgeon, a paediatric perfusionist, and a PICU team of two doctors and four nurses.

Each team member is responsible for and delivers their own aspect of paediatric cardiac surgery provision using the facilities and equipment available whilst working with their counterparts in the local hospital. Although hospitals that undertake adult cardiac surgery in lower and middle income countries are not usually able to provide the exact or optimum facilities required for paediatric cardiac surgery, it is possible to adapt an adult service safely enough to be able to operate on children as well. Deficiencies in equipment and other facilities as well as the near absence of congenital heart disease experience in the local team poses a huge challenge to the visiting team. This variance requires adaptability and flexibility from each team member who must call upon clinical skills acquired from working in developed health care systems where there is a strong emphasis on the basics of medicine and nursing. In addition, as the team is much smaller than the volunteers are used to working with back in their respective countries where there is a considerable pool of expertise to call upon in the case of clinically challenging patients, the smaller team must coalesce and support each other to help manage clinically fraught situations. Managing difficult clinical situations with a smaller pool of personnel and in resource constrained environments can potentially produce an uplifting of the clinical skills within the visiting team which may then translate into improved care for their local populations when they return.

The National Institute for Cardiac Outcomes Database (NICOR) in the UK, collects data and produces analysis to enable hospitals and healthcare improvement bodies to monitor and improve the quality of care and outcomes of cardiovascular patients (2). Examination of the database reveals that most paediatric cardiac centres in the UK, perform paediatric cardiac surgery on 6–8 children in a typical week.

We used the data derived from the NICOR database as a benchmark for the average number of cases performed per week in the UK and compared the activity of one international paediatric cardiac charity (Healing Little Hearts UK Registered Charity 1130194) with which the authors are associated, to describe our resource and time utilisation as the charity's operations are funded by public donations.

The number and type of cases performed between 1st April 2018 and 1st April 2019 were analysed using the charity database. Further analysis of the database explored the following: follow-up, costs of the trip, composition of the travelling teams, complications during the trips and mortality.

## Results

Between April 2018 and April 2019, Healing Little Hearts (HLH) undertook 23 international trips to countries including India, Malaysia, Nigeria, Romania, Tanzania, and several more.

In the 12 months, a total of 270 operations were performed which means that on average, 12 children were operated by HLH teams during each week the charity travelled which demonstrates a very efficient use of time, resources and donated funds.

As Table 1 highlights, the complexity of cases operated upon ranged from VSD closures, ASD closures, TOF repair and modified Blalock-Taussig shunt in neonates. The figures highlight that each surgeon and their supporting medical and nursing team are operating on and caring for significantly more children during this week compared to a typical week back in majority of paediatric cardiac centres in the UK. Crucially, all of this happens in resource limited settings. This amount of operating, provides the team with an excellent platform to teach and train the local teams. The teams usually work in adult setups both in theatre and in the intensive care unit, using equipment that is more suited for adult cardiac surgery. Often, surgeons with this charity undertake 2 week-long trips per year, operating on ~24 children during these trips. This is equivalent to 2 months operating per surgeon in many UK centres. Additionally, from the HLH database it is evident that the teams are comprised of doctors and nurses from across the developed world, i.e., all over the UK, parts of Europe, North America, and as far away as Australia. Many of these surgeons, nurses and physicians find these experiences so rewarding and beneficial that they travel numerous times in a year which in turn contributes to their own professional development. Again, with members of the team coming from across the globe, they bring a significant amount of varied experience, knowledge and expertise to the bedside which is shared with the local healthcare professionals and amongst the travelling team members who then become exposed to different ways of working clinically.

**Table 1:** List of surgical and interventional catheter procedures performed by the travelling team between 1st April 2018 and 1st April 2019.

**Number of procedures**	**Surgical procedures**	**Additional procedures**
81	VSD closure	
51	Tetralogy of Fallot repair	
46	ASD closure	
41	PDA closure	
7	TAPVR	
6	AVSD repair	
5	Glenn shunt	
4	Mitral valve replacement	
3	TAPVD	
1	Modified Blalock–Taussig shunt in neonate	
26		Non-surgical procedures (including PDA stents and ASD device closure)

## Follow-Up

HLH only works with hospitals that have a cardiologist who in turn coordinates the patients referred and organises follow up on children post-surgery. In addition, because HLH works with hospitals wishing to build capacity, the practise is usually to visit each partner hospital between two and four times per year which enables follow up on the children operated upon on previous visits.

## Team Composition

Teams are bespoke to the requirements of each hospital that HLH works with but in general the teams consist of 8–10 volunteers whose designations have already been described. A unique aspect of the service that HLH provides is to tailor each trip to the needs of each partner hospital. In other words, if a hospital has a surgeon and a cardiologist but no paediatric cardiac anaesthetist and PICU team then only those specific team members would constitute the HLH team.

## Costs Associated With the Trip

HLH costs are primarily related to the cost of flights needed to get the teams to the various hospitals that HLH works with. Each trip costs ~£6,000 and that money has been donated by the generous British public over the last 11 years. All HLH team members volunteer their time usually by taking annual leave and do not receive any remuneration for the clinical work that is done.

## Operating Plan During the Week

All the patients are screened thoroughly at a multidisciplinary meeting between the visiting team and the local team usually on the weekend and an operation list is finalised.

At the beginning of each week, the team operates on the simplest cases, increasing the complexity of the cases from Tuesday through to Thursday and then reducing the complexity on Friday. Extubation in theatre or soon thereafter is our standard approach although inevitably there is the odd patient or two still on the ventilator when the team leaves.

In these instances, HLH either tries to ensure that a member of the PICU team stays behind for a while longer or if that is not possible then we provide clinical support to the local team with help of internet based technology.

## Cost of Surgery

The host hospital's costs of cardiac surgery are recuperated from a combination of government funding and donations. It is essential that hospitals do not profit from the charity work that HLH does. Details of all patients operated upon by HLH are maintained on the charity's database. Equally, each partner hospital keeps its own database of patients operated upon.

## Complications

Out of the 270 cases performed by the team, 10 (4%) of the patients died post-operatively.

It is worth remembering that compared to the case load in higher income countries, the types of patients managed by travelling teams is entirely different from the patients back home as these patients usually present late with their clinical conditions, often as a result of adverse socioeconomic factors. Infective complications were more prevalent and postoperative sepsis is a significant factor causing mortality on these missions, something rarely seen in developed countries. Additional complications include unfortunate nursing errors such as potassium overdoses due to less stringent nursing checks as well as other drug errors and much slower reaction times to deteriorating patients due to the inexperience of local medical and nursing staff. In addition, due to the late diagnosis and presentation, a percentage of these children require longer stays in ICU and appear to have greater respiratory morbidity than observed in the UK such as ventilator associated pneumonias and pulmonary haemorrhage secondary to reperfusion injury. Pulmonary hypertension is also more prevalent in these settings due to the same confounding factors in these children.

## Discussion

CHD is a huge problem worldwide. Lack of access to care and insufficient expertise within the developing world to correct these defects results in vast numbers of children dying prematurely from eminently treatable cardiac conditions. The enormous global child health issue of untreated CHD has spawned the development of many charities from across the developed world that travel to low and middle-income countries to perform heart surgery on children (3–5). These projects are often looked at with great admiration, however, a key aspect which is often overlooked is the gradual empowerment of local medical and surgical teams. Firstly, let's explore the various challenges faced by the travelling healthcare teams during these endeavours.

### The Challenges

#### The Patient

The patients are often much more unwell than the cohort of patients looked after in the developed world as a result of delayed diagnoses and other co-morbidities of poverty. The late diagnoses are usually associated with significant structural changes to the patient's cardiorespiratory system which means that their pre- and post-operative course is often stormy.

#### The Environment

Resource-constrained and relatively alien working environments requires an upgrading of the ability and skills of each team member. The team can no longer rely on high-end technology and immediately available near-patient testing but instead they must adapt to a management method based on clinical skills and the first principles of medicine and nursing. The team often works within an adult ICU, usually with limited bed capacity, which requires considerable adaptation when managing paediatric cases. Resources so readily available back in the UK such as blood products are either not available or associated with considerable delay. In certain hospitals, cryoprecipitate is not available and often the easiest blood product to administer for postoperative bleeding is whole blood which can be associated with significant pro- inflammatory effects and subsequent morbidity.

#### The Operating Room and the ICU

Travelling surgeons are usually having to use adult cardiac surgical instruments for very delicate operative procedures. Anaesthetists must successfully anaesthetise and ventilate the patients using machines not entirely suitable for children. Similarly, in the ICU, the team must adapt to adult ventilators. Whilst these challenges result in difficult and testing times for the travelling teams, they transform into unique learning experiences which the teams take back with them to their respective countries.

### The Benefits

#### The Local Team

The local team of doctors and nurses have often had no prior training or experience in paediatric intensive care or paediatric cardiology. Consequently, they are often unfamiliar with normal physiological parameters for children, let alone the complexity and nuances of the various cardiac cases operated upon. To combat this problem, the travelling team provides constant education to the local teams to boost their knowledge and skills when managing complex paediatric cases. Examples of this include short but informative daily tutorials held by the travelling doctors and nurses on important topics such as drug administration and vital observations. Additionally, a mentorship scheme is established wherein the lead travelling surgeon will train a local surgeon for the entire week in theatre to boost his/her skills, competence and confidence. This example of pedagogy has ultimately led to two paediatric cardiac services within India, previously with minimal experience, being signed off as self-sufficient and able to perform life-saving cardiac surgery without assistance from travelling teams. It is a strategy that potentially bodes well for the future with the goal to make all collaborating cardiac centres self-sufficient through this global initiative. Greater cardiac surgical activity within the developing world leads to a reduction in the number of children tragically dying due to these correctable defects.

#### The Travelling Team

New experiences, challenging cases and suboptimal environments provide a positively unique opportunity for each member of the travelling team. These altruistic acts are undoubtedly extremely satisfying and rewarding. In addition, as the entire volunteer team has cared for a much larger number of cases in a typical week than they would do in their own units, it could translate into enhanced expertise within each member of the team. Based on the above comments, we propose that a surgeon who has operated on an average of 12 children in a week may return home with his or her operative skills finely honed and that would hopefully produce better outcomes for patients back at home. The same analogy is applicable to all members of the travelling teams. Beyond the clinical realm, it is worth stating that each visit by a volunteer to a foreign country represents a life experience that they would not necessarily have had otherwise nor indeed even contemplated.

## The Future

It is all too easy to label such missions as charity endeavours which unfortunately misses the crucial point which is that the recipient healthcare teams and the children operated upon benefit immensely from such collaborations. The children are given a new lease of life through these operations. Through the continuous training and teaching that occurs during these missions, it gradually builds up confidence, knowledge and expertise within the local teams such that they can become self-sufficient. With this being the ultimate goal in mind, this rewarding project can have a domino-like effect and translate into increasing numbers of hospitals, previously ill-equipped to treat paediatric cardiac patients, becoming trained in doing so. The world is now a much smaller place which lends itself very nicely to such rewarding, life saving, and mutually beneficial partnerships.

Underpinned by robust data collection along with stringent risk assessment and strict governance arrangements of the work carried out by visiting international teams, there is no reason why such medical relationships should not be formalised. In a globalised world, trade links between countries could become the template for the sharing of good medical practise across the globe. Using either ongoing trade and commerce relationships or previous historical ties such as the Commonwealth as a template, health partnerships should be established whereby governments of higher income countries should seek to work with governments of lower and middle income counties to help build capacity, i.e., train and empower local doctors and nurses to carry out and perform the entire range of paediatric cardiac surgery through multiple visits over a medium term collaboration of 5–10 years. These partnerships will be a win-win situation whereby both the teams from higher income countries and the hospitals and children in lower income countries can benefit. Continuous training and education provision for local teams within the developing world can result in increasing numbers of cardiac centres becoming self-sufficient and able to provide cardiac care without additional support. Goal 3 of the United Nations Sustainable Development Goals 2015 is to reduce the staggering 6 million childhood deaths in children <5 years occurring annually of which CHD comprises a significant proportion (6, 7).

The only way that can be achieved is if managing the health of children in our ever-shrinking world is no longer constrained by geographical borders but instead is guided by need and delivered by such partnerships.

## Limitations

Our study and the subsequent discussion attempts to explore the potential benefits of international charity work for both the travelling, but more importantly, the local healthcare teams by identifying the challenges and solutions to overcome them. Whilst the aforementioned points provide strong arguments outlining these positive mutual effects, we appreciate and recognise the lack of robust and conclusive data to support such conclusions. In order to address this, future qualitative analysis including distribution of surveys to both the travelling and local team members soliciting their opinions on how these missions positively impact their skills and expertise would be warranted.

## Conclusion

A burgeoning amount of literature exists about the catastrophic lack of resources available in many parts of the developing world for paediatric cardiac surgery and the benefit that accrues to the children in the poorest countries because of the charitable visits. However, there has been virtually no evaluation of the considerable and mutual benefits to both the visiting and more importantly the local teams during these humanitarian endeavours. The authors hope that such observations provide a template for the formalising of such mutually beneficial and life-saving collaborations between higher and lower and middle income countries. It is our duty to share our world-renowned expertise and skills with these less fortunate countries to save lives on an international scale.

## Author Contributions

All authors listed have made a substantial, direct and intellectual contribution to the work, and approved it for publication.

## Conflict of Interest

The authors declare that the research was conducted in the absence of any commercial or financial relationships that could be construed as a potential conflict of interest.
